# Functional and topological characterization of transcriptional cooperativity in yeast

**DOI:** 10.1186/1756-0500-5-227

**Published:** 2012-05-10

**Authors:** Daniel Aguilar, Baldo Oliva

**Affiliations:** 1 Structural Bioinformatics Group (GRIB/IMIM), Departament de Ciencies Experimentals i de la Salut, Universitat Pompeu Fabra, Barcelona Biomedical Research Park (PRBB), c/Dr. Aiguader 88, 08003, Barcelona, Spain

**Keywords:** Regulatory network, Transcription factor, Gene regulation, Gene expression, Transcriptional cooperativity

## Abstract

**Background:**

Many cellular programs are regulated through the integration of specific transcriptional signals originated from external stimuli, being cooperation between transcription factors a key feature in this process. In this work, we studied how transcriptional cooperativity in yeast is aimed at integrating different regulatory inputs rather than controlling particular cellular functions from a organizational, evolutionary and functional point of view.

**Findings:**

Our results showed that cooperative transcription factor pairs co-evolve and are essential for the life of the cell. When organized into a layered regulatory network, we observed that cooperative transcription factors were preferentially placed in the middle layers, which highlights a role in regulatory signal integration. We also observed significant co-activity and co-evolution between members of the same cooperative pairs, but a lack of common co-expression profile.

**Conclusions:**

Our results suggest that transcriptional cooperativity has a specific role within the regulatory control scheme of the cell, focused in the amplification and integration of cellular signals rather than control of particular cellular functions. This information can be used for better characterization of regulatory interactions between transcription factors, aimed at determining the spatial and temporal control of gene expression.

## Background

Many cellular programs are regulated through the integration of specific transcriptional signals originated from external stimuli. In order to understand these programs, it is necessary to explore modes of interaction between transcription factors (TFs) such as transcriptional cooperativity. Particularly in eukaryotes, the process of cooperativity enables a small number of TFs to combine their regulatory influences to execute a large number of regulatory decisions [1,2]. This can be achieved through different mechanisms, such as interaction between adjacent TFs on the promoter, interaction between distantly located cis-regulatory elements or even through mechanisms devoid of protein contacts [3-6].

Previous studies have devised methods for computationally detecting and measuring transcriptional cooperativity based on different mechanisms of cooperation such as co-expression, co-binding to the promoter or TF-TF interaction. These works produced different lists of cooperative transcription factor pairs (CTFPs) [3-9]. In a previous work we found that a core of the predicted CTFPs actually shared some particular characteristics when analyzed in terms of their placement in the protein interaction network and in the regulatory network [10].

In this work, we characterize transcriptional cooperativity from a functional and evolutionary point of view. Also, we explore how transcriptional cooperativity in yeast is aimed at integrating different regulatory inputs and the transmission of the regulatory signal to other TFs which control particular cellular functions [10,11]. To do so, we examined the role of known CTFPs in the hierarchical layout of the regulatory network.

## Findings

### Are cooperative TFs essential for the life of the cell?

We observed that the probability that both members of a CTFP are synthetic lethals is 144-fold larger than for random expectation, which is statistically significant (Fisher's test; *p*-value = 1.63*10^-6^). For comparison, TF pairs regulating similar functions (i.e. co-functional, see Methods) were synthetic lethals 4.74 times more often than random expectation. This suggests that the deletion of a member of a cooperative TF might disturb the transcriptional profiles of some genes but renders the cell viable. The deletion of both members of a CTFP, however, is critical. This highlights the importance of cooperativity as a transcriptional coordinative process.

### Do cooperative TFs co-evolve?

It is known that essential TFs tend to evolve slower that non-essential ones [12]. Furthermore, it is known that interacting proteins pairs are likely to co-evolve [13,14]. These observations made us wonder whether CTFPs shared similar selection processes. We found a positive correlation between the protein evolution rate of members of the same CTFP (Spearman's test; r = 0.464, *p*-value < 2.2*10^-16^). This suggests that mutations in only one of the members of a CTFP are enough to have a deleterious effect and suffer from a negative selection, which highlights the functional dependence between both members of a CTFP. This result is interesting because only 5 of the 32 CTFPs in our set (16%) are known to physically interact. We have to note that co-evolution does not imply a particular substitution rate along evolution, but that both members of the same CTFP evolve at a similar rate (in fact, we did not observe a significant trend for CTFPs towards a preferential evolutionary rate). If we assume that the known yeast interactome comprehensively covers the protein-protein interactions between transcription factors, the value of this observation lies in the fact that the rate of evolution of a TF is influenced by its cooperative interactions, regardless of a physical interaction between them.

### Are cooperative TFs co-expressed or co-active?

We did not observe any significant correlation between the mRNA expression profiles of the members of the same CTFP in the different cellular conditions under study (see Methods). We did not did find any significant correlation either between expression levels (number of copies/cell), half-lives of the transcripts or transcriptional frequency for members of the same CTFP. This lack of correlation may be explained by the presence of post-transcriptional mechanisms regulating the functional activation of TFs and by the average window of activity of TFs inside the cell. We then investigated whether the activity profiles of the members of the same CTFP were correlated (since the actual activity of a TF can be uncoupled from its expression profile [15]). We did find a significant correlation between the activities of TFs belonging in the same CTFP for 15 of the 17 experiments (Table 1). This suggests that post-transcriptional modifications have a stronger influence than expression regulation in transcriptional cooperativity. We also examined co-activity in a layer-by-layer basis (Additional file 1). Finally, we measured the correlation between evolutionary rate and expression levels (copies/cell) for members of the same CTFP. We observed a strong anti-correlation of ρ = −0.72 (Spearman's test; *p*-value = 0.021). These results agree with previous observations [16,17].

**Table 1:** Co-activity between CTFPs

**Condition**	**Average co-activity**	**Co-activity increase (in *n* -fold)**	** *p* -value**
**Anearobic N-C-P-S chemostats**	0.58	1.63	8.99*10^-3^
**C-S-P-N chemostat limitation**	0.49	1.25	4.73*10^-2^
**Calcineurin**	0.55	2.48	9.13*10^-7^
**Carbon-limited chemostats**	0.56	1.37	6.97*10^-3^
**Cell Cycle**	0.43	6.39	2.99*10^-9^
**Cold shift**	0.53	1.20	3.66*10^-2^
**Compounds and stress**	0.39	3.51	9.17*10^-9^
**DNA damage**	0.41	2.69	9.50*10^-7^
**Environmental stress**	0.42	3.64	9.38*10^-9^
**Lithium response**	0.56	1.29	2.34*10^-1^
**Map kinase**	0.34	2.12	1.62*10^-4^
**Proteasome inhibitor**	0.38	3.61	1.29*10^-8^
**Regulation by PDR1**	0.27	1.72	2.32*10^-1^
**Rosetta compendium**	0.37	4.24	2.09*10^-9^
**Sporulation**	0.27	1.60	1.91*10^-2^
**TCA cycle mutants**	0.28	2.46	5.41*10^-6^
**Titratable promoter alleles**	0.41	4.41	3.57*10^-9^

Average co-activity (calculated using a squared Spearman's correlation coefficient) between members of the same CTFP. Increase in co-activity is calculated as the ratio of average correlation in CTFPs vs average correlation in 1000 non-cooperative TF pairs.

### Analysis of the regulatory hierarchy

**Figure 1:**
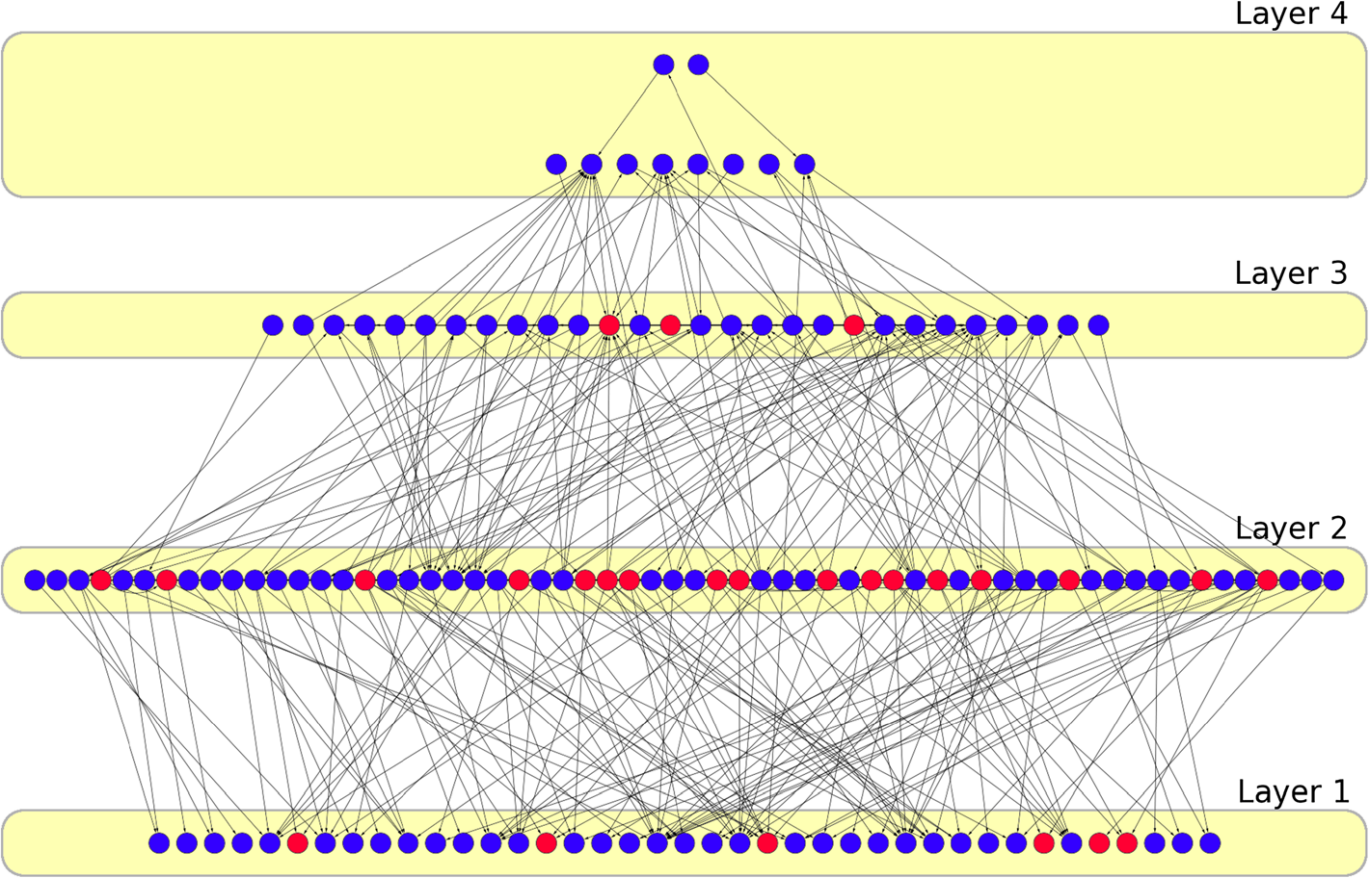
**Regulatory hierarchy.** TFs are represented as nodes and regulatory interactions as directed edges. The two topmost layers were merged for the analysis. Blue nodes: non-cooperative TFs. Red nodes: cooperative TFs.

It is known that the transcriptional regulatory network has a multi-layered hierarchical structure acting as a decision-making system (Figure 1), where the topmost layers is where external stimuli reach the regulatory network (e.g. through a signalling cascade) [11]. The regulatory signal is then integrated with other signals and amplified as it travels down the hierarchy through the middle layers (which act as bottlenecks in the information flow). In response, TFs in the lower layer execute the transcriptional response by turning on/off the activity of different groups of genes. This is known as the cogitation process.

### Is cooperativity associated with the cogitation process?

Because members of the same CTFP do not share similar regulatory inputs, we suggested in a previous work that cooperativity might have a role in integrating multiple regulatory signals [10]. If cooperativity provides integration and amplification of regulatory signals, then CTFPs should be preferentially placed in the middle layers of the hierarchy, since this is where incoming signals from the global modulators are integrated before the activation of the different sets of genes which carry out particular functions.

We found that CTFPs were slightly (but significantly) under-represented in the layer-1 of the hierarchy (odds ratio = 0.75; *p*-value = 1.17*10^-4^), and were clearly over-represented in the layer-2 (odds ratio = 5.1; *p*-value = 1.73*10^-7^). The presence of cooperative TFs in layer-1 may be surprising because TFs in layer-1 only control non-TF genes. However, we have to take into account that cooperation between TFs is not necessarily dependent on regulation of expression, but may be based on co-binding to the promoter [7] or in protein-protein interactions [6]. This explains the presence of cooperative TFs in this layer.

These findings confirm that cooperativity is mainly related not to the process of execution of transcriptional responses (if so, CTFPs would be preferentially placed in layer-1), nor to the reception of external stimuli (their presence is not relevant in the upper layer). Instead, cooperativity seems to be associated mainly with the integration of regulatory signals and their transmission to the lower layers.

We also found that the protein functions regulated by cooperative TFs in the layer-1 were significantly enriched in metabolism-related functions such as *Metabolism*, *Regulation of metabolism and protein function* or *Cellular transport* (Table 2). However, other housekeeping functions (e.g. cell-cycle-related and communication-related) were under-represented. This suggests that metabolism is the cellular function to be cooperatively regulated at the bottom level of the hierarchy. Conversely, cooperative TFs in layer-2 were responsible of cell-cycle related functions, which is consistent with a role in coordination of broad cellular processes. Also, the function *Interaction with the environment* is significantly over-represented, thus implying that cooperative TFs in this level are responsible for passing external signals down to the lower layers of the hierarchy. Finally, although no communication-related functions were significantly over-represented in layer-3, we observed a clear under-representation of housekeeping functions (such as metabolism), preferentially regulated in the bottom layer. The same analysis using Gene Ontology terms instead of FunCat categories yielded very similar results (Additional file 2).

**Table 2:** Functional enrichment in the regulatory hierarchy

	**Function**	** *z* -score**	** *p* -value**
**Layer-1**	Biogenesis of cellular components	−2.26	0.02
	Cell cycle and DNA processing	−2.29	0.01
	Cell rescue, defense and virulence	−4.16	1.58*10^-5^
	Cell type differentiation	−3.28	5.18*10^-4^
	Cellular communication/signal transduction mechanism	−2.1	0.01
	Cellular transport, transport facilities and transport routes	6.56	2.74*10^-11^
	Interaction with the environment	−3.93	4.19*10^-5^
	Metabolism	4.67	1.51*10^-6^
	Protein with binding function or cofactor requirement	−2.8	2.56*10^-3^
	Regulation of metabolism and protein function	6.1	5.47*10^-10^
**Layer-2**	Cell type differentiation	1.95	0.02
	Cellular transport, transport facilities and transport routes	−2.18	0.01
	Interaction with the environment	2.28	0.01
**Layer-3**	Metabolism	−1.95	0.02
	Regulation of metabolism and protein function	−1.88	0.03
	Cell type differentiation	−2.18	0.01
	Interaction with the environment	−2.62	4.43*10^-3^
	Regulation of metabolism and protein function	−1.81	0.03

This table shows all significantly over-represented or under-represented functional categories for cooperative TFs in regulatory hierarchy. Negative *z*-scores mean under-representation, positive *z*-scores mean over-representation.

## Conclusions

In this work, we studied the role of transcriptional cooperativity in the control of regulatory programs in yeast. Our results suggest that transcriptional cooperativity has a specific role focused in the amplification and integration of cellular signals rather than control of particular sets of genes or detection of external stimuli. We also show a functional dependence between members of a cooperative TF pair (both are synthetic lethals, co-evolve and have similar activity profiles). This information can be used for better characterization of regulatory interactions between transcription factors, aimed at determining the spatial and temporal control of gene expression.

## Methods

Associations between TFs and target genes were extracted from Beyer *et al.*[18]. We used the subset of TF-regulated gene associations labeled as *highly confident* by the authors. We built a set of CTFPs based on the compilation of computationally-predicted CTFPs by four different methods [6-10]. We selected those TF pairs predicted as cooperative at least by two methods. The resulting amount of CTFPs was 32, composed by the pairing of 26 distinct TFs (Additional file 3).

Following the Breadth-First Search algorithm described by Yu & Gerstein [11], we built a directed network of TFs as a multi-layered hierarchical structure (Figure 1). We merged the two upper layers of the network (with 8 and 2 TFs, respectively) in order to avoid a low number of TFs that would hinder statistical calculations. The final network had four layers (the bottom layer termed *layer-1*, the topmost layer termed *layer-4*) composed by 148 TFs and 96 regulatory interactions (Additional file 4). This network will be referred to as *regulatory hierarchy*. A slightly different implementation of the algorithm places all targets of a TF in the same level, thus forcing all interactions in the hierarchy to point downward or horizontally, but never upwards [19]. We also built and analyzed this hierarchy (Additional file 5, Additional file 6). The enrichment in CTFPs for level *n* was calculated as the ratio of the probability of finding a CTFP in that level vs random expectation. The statistical significance was calculated using 10 [5] random hierarchies where the target genes were randomly exchanged between TFs.

We obtained mRNA expression data for the following cellular conditions: diauxic shift [20], cell cycle [21], sporulation [22], and six environmental stress conditions: heat, acid, alkali, peroxide, NaCl and sorbitol [23]. Expression levels (copies/cell), apparent half-life of the transcripts (in minutes) and transcriptional frequency (mRNAs/hour) were obtained from Holstege *et al*[24]. Correlation between expression levels was calculated using a Spearman's correlation test. We downloaded the protein activity profiles of the TFs in our sets from 17 experiments of the database RegulonProfiler, where TF activity profiles are inferred from genomewide changes in mRNA expression patterns of groups of genes with similar regulation (called ChIP-based regulons), which allowed the authors to quantify the post-translational activity of TFs [25]. Only activity profiles with E-value < 0.05 were considered. Correlations were calculated using a squared Spearman's correlation test [26,27]. In all cases, the distribution of the correlation of the activity levels of CTFPs was compared against the distribution of the activity levels of 1000 non-CTFPs by means of a KS test.

Information on the essentiality of yeast proteins was downloaded from the Yeast Deletion Project [28]. Information on synthetic lethals was obtained from the BioGrid database [29]. Association between essentiality and transcriptional cooperativity was calculated by means of a Fisher's test.

Protein evolutionary rate for TFs was obtained from Xia *et al*[30]. The correlation between evolutionary rates and expression levels for CTFPs was calculated as the dn/ds ratio for members of the same CTFP and the ratio of their expression levels. A Spearman's test was used to calculate the correlation.

Protein functions were extracted from the FunCat catalogue [31]. Being FunCat a hierarchical classification, we used first-level functions with experimental evidence, which amounted to 16 different functions. Functional similarity between TFs was calculated as in Aguilar & Oliva [10]. We defined two TFs as co-functional if their functional similarity was larger than the 90^th^ percentile of the distribution of the functional similarity values for all TF pairs.

We first measured the enrichment for each function in each level of the regulatory hierarchy by means of a *z*-score, using a random model consisting in 10 [5] regulatory hierarchies where the gene functions controlled by the TFs were randomly exchanged. We repeated this analysis using the Gene Ontology functional annotation at depth level 2 (which is roughly equivalent to the first level of FunCat) [32]. TFs annotated at lower levels were re-annotated with the corresponding parent terms of level 2. Only experimental annotations were used. The R software was used for all statistical tests [33].

### Availability of supporting data

The data sets supporting the results of this article are included within the article and its additional files.

## Abbreviations

TF, Transcription factor; CTFP, Cooperative transcription factor pair; KS, Kolmogorov-Smirnov.

## Competing interests

The authors declare that they have no competing interests.

## Authors' contributions

DA conceived the study and carried out the analysis. BO participated in the design of the study and helped to draft the manuscript. Both authors read and approved the final manuscript.

## Supplementary Material

Additional file 1 **Co-activity between CTFPs in the regulatory hierarchy.** Average co-activity (calculated using squared Spearman's correlation coefficient) between members of the same CTFP in the different layers of the regulatory hierarchy. Click here for file

Additional file 2 **Functional enrichment in the regulatory hierarchy in Gene Ontology terms.** This document contains a table with the functional enrichment in the regulatory hierarchy in Gene Ontology terms. Click here for file

Additional file 3 **List of cooperative TF pairs.** This file contains the list of cooperative TF pairs. Click here for file

Additional file 4 **Regulatory hierarchy.** This file contains the regulatory hierarchy. Click here for file

Additional file 5 **Regulatory hierarchy without upwards regulatory interactions.** This file contains the regulatory hierarchy with no upwards regulatory interactions. Click here for file

Additional file 6 **Regulatory hierarchy without upwards regulatory interactions.** This document explains the methods used to build this hierarchy and the results of its analysis. Click here for file
