# Supplementary material for: Functional and topological characterization of transcriptional cooperativity in yeast
Source: BMC Res Notes. 2012 May 10;5:227. doi: 10.1186/1756-0500-5-227 (PMC3499397; doi:10.1186/1756-0500-5-227)
Supplement: Additional file 1 — Co-activity between CTFPs in the regulatory hierarchy. Average co-activity (calculated using squared Spearman's correlation coefficient) between members of the same CTFP in the different layers of the regulatory hierarchy. [file 1756-0500-5-227-S1.pdf]

Additional file 1. Co-activity between CTFPs in the regulatory hierarchy

Average co-activity (calculated using squared Spearman's correlation coefficient) between members of the same CTFP in the different layers of the regulatory hierarchy. Increase in co-activity is calculated as the ratio of average correlation in CTFPs vs average correlation in 1000 non-cooperative TF pairs. White rows indicate statistically significant results ($\alpha < 0.05$). Grey rows indicate non-significant results. Layers 3 and 4 are not present because there are no CTFPs in those layers (although cooperative TFs are present, they cooperate with TFs in other layers). The *p*-values are, on average, larger than in table 1 because of the smaller size of the distributions and KS test is affected by sample size (this is, in table 1 all CTFPs were used to built de distribution of co-activity values; in this table, only CTFPs of a single layer are used).

For layer-1, we observed significant co-activation of CTFPs only for 9 of the 17 experiments. If we take into account that cooperative TFs are under-represented in this layer (see manuscript), the lack of a large cooperative response is not surprising. Also, some of the experiments (e.g. *Anearobic N-C-P-S chemostats*, *C-S-P-N chemostat limitation, Cold shift*) trigger the response of a large number of genes (52%, 31%, 25% of the genes in yeast genome, respectively; Tai *et al.*, 2005; Boer *et al.*, 2002; Sahara *et al.*, 2002). Such a broad response is likely to be controlled by cooperative and non-cooperative TFs alike. Finally, the *Regulation by PDR1* experiment did not trigger any significant co-activity in cooperative TFs in layer-1 since PDR1 (YGL013C) is a TF placed in layer-2.

As for layer-2, a significant co-activity increase in this layer is suggestive of the need of cooperatively-regulated signal amplification when carrying out the response to a stimulus. The fact that the experiments *Anearobic N-C-P-S chemostats*, *C-S-P-N chemostat limitation* and *Cold shift* did not trigger the activity of CTFPs neither on layer-1 nor in layer-2 may mean the lack of a cooperatively-regulated signal transmission process for this kind of experiments which affect cell growth. In other experiments which are known to depend of signal amplification and coordination (such a *Cell cycle* and *Environmental stress*), we observed significant increase in co-activity in this layer.

Taken together, these results suggest that the co-activation of CTFPs is a process that takes place across all layers of the regulatory hierarchy for most experimental conditions. This is so because experimental perturbations reach the hierarchy through the topmost layers as external signals, and then travel downwards to activate/deactivate the expression of particular sets of genes.

	Layer-1			Layer-2		
	Average co-activity	Co-activity variation	*p*-value	Average co-activity	Co-activity variation	*p*-value
**Anearobic N-C-P-S chemostats**	0.76	2.15	2.15E-001	0.61	1.69	1.47E-002
**C-S-P-N chemostat limitation**	0.34	1.01	8.60E-001	0.59	1.42	5.95E-002
**Calcineurin**	0.70	4.15	2.86E-002	0.60	2.81	2.72E-007
**Carbon-limited chemostats**	0.76	1.68	3.22E-001	0.58	1.37	1.20E-001
**Cell Cycle**	0.78	7.20	2.12E-002	0.43	6.42	2.39E-007
**Cold shift**	0.70	1.50	6.21E-001	0.53	1.23	5.02E-001
**Compounds and stress**	0.63	4.04	2.12E-002	0.48	4.28	1.81E-010
**DNA damage**	0.70	3.66	2.86E-002	0.43	3.01	1.52E-007
**Environmental stress**	0.69	6.65	2.12E-002	0.46	4.14	2.94E-008
**Lithium response**	0.44	1.35	9.20E-001	0.65	1.53	9.32E-002
**Map kinase**	0.73	4.25	2.86E-002	0.35	2.49	9.34E-005
**Proteasome inhibitor**	0.64	4.57	2.12E-002	0.31	2.93	2.73E-004
**Regulation by PDR1**	0.58	3.29	2.65E-001	0.32	1.62	4.14E-002
**Rosetta compendium**	0.78	5.41	2.12E-002	0.39	5.46	4.54E-011
**Sporulation**	0.43	2.32	1.09E-001	0.35	1.66	1.34E-002
**TCA cycle mutants**	0.60	3.62	2.65E-001	0.21	1.90	8.70E-003
**Titratable promoter alleles**	0.80	6.12	2.12E-002	0.42	5.29	1.63E-008

REFERENCES

Boer VM, de Winde JH, Pronk JT, Piper MD. The genome-wide transcriptional responses of Saccharomyces cerevisiae grown on glucose in aerobic chemostat cultures limited for carbon, nitrogen, phosphorus, or sulfur. J Biol Chem. 2003 Jan 31;278(5):3265-74. Epub 2002 Oct 31

Sahara T, Goda T, Ohgiya S. Comprehensive expression analysis of time-dependent genetic responses in yeast cells to low temperature. J Biol Chem.2002 Dec 20;277(51):50015-21. Epub 2002 Oct 11

Tai SL, Boer VM, Daran-Lapujade P, Walsh MC, de Winde JH, Daran JM, Pronk JT. Two-dimensional transcriptome analysis in chemostat cultures. Combinatorial effects of oxygen availability and macronutrient limitation in Saccharomyces cerevisiae. J Biol Chem. 2005 Jan 7;280(1):437-47
