# Supplementary material for: Functional and topological characterization of transcriptional cooperativity in yeast
Source: BMC Res Notes. 2012 May 10;5:227. doi: 10.1186/1756-0500-5-227 (PMC3499397; doi:10.1186/1756-0500-5-227)
Supplement: Additional file 2 — Functional enrichment in the regulatory hierarchy in Gene Ontology terms. This document contains a table with the functional enrichment in the regulatory hierarchy in Gene Ontology terms. [file 1756-0500-5-227-S2.pdf]

Additional file 2. Functional enrichment in the regulatory hierarchy in Gene Ontology terms

This table shows all significantly over-presented or under-represented Gene Ontology terms for cooperative TFs in regulatory hierarchy. Only Gene Ontology terms of depth 2 were used. Negative *z*-scores mean under-representation (grey cells), positive *z*-scores mean over-representation. Protein functions regulated by cooperative TFs in the layer-1 are significantly enriched in metabolism-related functions such as *Cellular metabolic process*, *Primary metabolic process* or *Small molecular metabolic process*. However, cell-cycle-related and communication-related functions (e.g. *Cell cycle process*, *Response to external stimulus*) were significantly under-represented. This agrees with results shown in table 2 in the main text. Cooperative TFs in layer-2, despite nor being significantly enriched in functions linked to cell cycle control, are clearly not related to cell communication functions. The only exception is the function *Macromolecule metabolic process*, which is over-represented in layer-2 and under-represented in layer-1 (and for which there is no equivalent category in FunCat). Its placement in this layer can be explained by its broad function comprising biosynthesis and modification of proteins as well as gene expression regulation as a whole, thus affecting a large number of genes. Layer-3 shows an almost inverse distribution of protein functions with respect to the bottom layer. It is enriched in a variety of functions related to cellular communication and response to external stimuli but housekeeping cellular activities are significantly under-represented, which agrees with the role of this layer in signal detection and downward transmission.

	Function	z-score	p-value
Layer-1	biosynthetic process	-2.59	$4.82*10^{-3}$
	response to stress	-3.24	$6.01*10^{-4}$
	cell communication	-3.04	$1.19*10^{-3}$
	response to external stimulus	-3.04	$1.19*10^{-3}$
	cellular component organization or biogenesis at cellular level	-2.87	$2.03*10^{-3}$
	cellular component organization	-2.87	$2.03*10^{-3}$
	cell cycle process	-2.27	$1.15*10^{-2}$
	macromolecule metabolic process	-3.3	$4.90*10^{-4}$
	filamentous growth	-2.13	$1.67*10^{-2}$
	response to abiotic stimulus	-2.1	$1.79*10^{-2}$
	cellular response to stimulus	2.27	$1.16*10^{-2}$
	cellular metabolic process	3.3	$4.87*10^{-4}$
	primary metabolic process	3.4	$3.37*10^{-4}$
	nitrogen compound metabolic process	4.44	$4.52*10^{-6}$
	small molecule metabolic process	5.41	$3.10*10^{-8}$
Layer-2	cell communication	-3.27	$5.43*10^{-4}$
	response to external stimulus	-3.27	$5.43*10^{-4}$
	cellular response to stimulus	-2.69	$3.55*10^{-3}$
	macromolecule metabolic process	1.9	$2.90*10^{-2}$
Layer-3	primary metabolic process	-2.87	$2.03*10^{-3}$
	cellular metabolic process	-2.78	$2.72*10^{-3}$
	nitrogen compound metabolic process	-2.37	$8.88*10^{-3}$
	macromolecule metabolic process	-2.03	$2.14*10^{-2}$
	small molecule metabolic process	-1.79	$3.69*10^{-2}$
	response to abiotic stimulus	2.45	$7.09*10^{-3}$
	regulation of biological process	3.02	$1.27*10^{-3}$
	cellular component organization or biogenesis at cellular level	3.35	$4.05*10^{-4}$
	cellular component organization	3.35	$4.05*10^{-4}$
	response to chemical stimulus	3.88	$5.23*10^{-5}$
	aging	5.16	$1.24*10^{-7}$
	cell aging	5.16	$1.24*10^{-7}$
	cellular response to stimulus	5.92	$1.57*10^{-9}$
	response to stress	6.76	$7.09*10^{-12}$
	cell communication	14.32	$8.33*10^{-47}$
	response to external stimulus	14.32	$8.33*10^{-47}$
