# Supplementary material for: Functional and topological characterization of transcriptional cooperativity in yeast
Source: BMC Res Notes. 2012 May 10;5:227. doi: 10.1186/1756-0500-5-227 (PMC3499397; doi:10.1186/1756-0500-5-227)
Supplement: Additional file 6 — Regulatory hierarchy without upwards regulatory interactions. This document explains the methods used to build this hierarchy and the results of its analysis. [file 1756-0500-5-227-S6.pdf]

Additional file 6. Regulatory hierarchy without upward regulatory interactions

This document explains the methods used to build this hierarchy and the results of its analysis.

Methods

Following the Breadth-First Search algorithm as described by Bhardwaj[1], we built a directed network of TFs where no upward regulatory interactions were possible. The final hierarchy had three layers (the bottom layer termed *layer-1*, the topmost layer termed *layer-3*) composed by 150 TFs and 71 regulatory interactions (figure S1). Despite the absence of upwards interactions, the shape of this hierarchy is also diamond-like, as is the case of the *regulatory hierarchy* in the main manuscript (hereinafter referred to simply as *regulatory hierarchy*). This suggests that not method used to built the hierarchy but the data source is responsible for this shape.

Results and discussion

Results for this hierarchy were similar to those obtained for the *regulatory hierarchy* in the main manuscript.

Is cooperativity related to the cogitation process?

We found that cooperative TF pairs were under-represented in the layer-1 of this hierarchy (odds ratio = 0.77; Fisher's test, *p*-value = $8.85*10^{-6}$), and were significantly over-represented in the layer-2, although the odds ratio was not as large as for the *regulatory hierarchy* (odds ratio = 1.84; *p*-value = $2.9*10^{-4}$). Our results show that cooperative TFs do not have a higher betweenness than non-cooperative ones (KS test, *p*-value = 0.273)

Do cooperative TFs control the same cellular functions?

Table S1 shows all significantly over-presented or infra-represented functional categories (as *z*-scores) for cooperative TFs in the regulatory hierarchy without upward regulation. The distribution of functions in this hierarchy is mainly characterized by the over-representation of the function *Cell rescue, defense and virulence* in the layer-2 of the hierarchy, which

reinforces the role for the middle layer in the response of external stimuli (in the *regulatory hierarchy*, only the related function *Interaction with the environment* was over-represented). The upper layer is devoid of over-represented functions, which might be a consequence of the low count of TFs in this layer.

References

1. Bhardwaj N, Kim PM, Gerstein MB: Rewiring of transcriptional regulatory **networks: hierarchy, rather than connectivity, better reflects the importance of regulators**. *Sci Signal*. 2010, **3**: ra79.

Figures

Figure S1. Regulatory hierarchy without upward regulation. TFs are represented as nodes and regulatory interactions as edges. The two topmost layers were merged for the analysis. Blue nodes: non-cooperative TFs. Orange nodes: cooperative TFs.

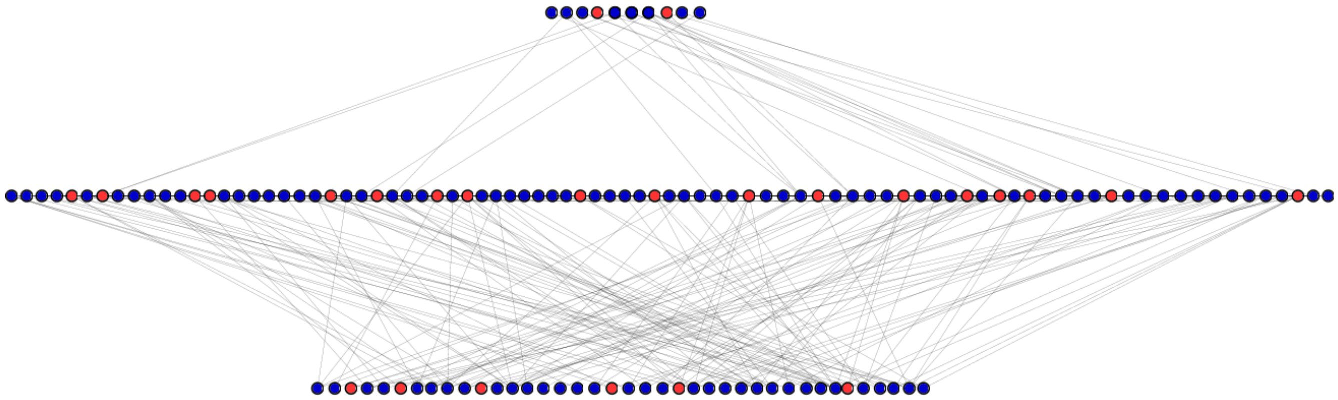

Tables

Table S1. Significantly over-presented or infra-represented functional categories (as *z*-scores) for cooperative TFs in the regulatory hierarchy without upward regulation. Grey background indicates that the function was not observed for the *regulatory hierarchy* in the main manuscript.

	**Function**	***z*-score**	***p*-value**
**Layer-1**	Biogenesis of cellular components	-2.23	0.01
	Cell cycle and DNA processing	-2.23	0.01
	Cell rescue, defense and virulence	-4.16	$1.56*10^{-5}$
	Cell type differentiation	-3.30	$4.82*10^{-4}$
	Cellular communication/signal transduction mechanism	-2.09	0.01
	Cellular transport, transport facilities and transport routes	6.57	$2.50*10^{-11}$
	Interaction with the environment	-3.81	$7.02*10^{-5}$
	Metabolism	4.65	$1.66*10^{-6}$
	Protein with binding function or cofactor requirement	-2.74	$3.04*10^{-3}$
	Regulation of metabolism and protein function	6.44	$5.81*10^{-11}$
**Layer-2**	Cell rescue, defense and virulence	2.96	$1.56*10^{-3}$
	Cell type differentiation	2.32	0.01
	Cellular transport, transport facilities and transport routes	-3.16	$8.00*10^{-4}$
	Interaction with the environment	2.71	$3.36*10^{-3}$
	Protein with binding function or cofactor requirement	2.4	$8.11*10^{-3}$
	Regulation of metabolism and protein function	-2.92	$1.76*10^{-3}$
**Layer-3**	Cell rescue, defense and virulence	-1.77	0.03
	Metabolism	-3.20	$6.88*10^{-4}$
	Protein with binding function or cofactor requirement	-2.30	0.01
